# Influence of *Rhizoctonia solani* and *Trichoderma* spp. in growth of bean (*Phaseolus vulgaris* L.) and in the induction of plant defense-related genes

**DOI:** 10.3389/fpls.2015.00685

**Published:** 2015-09-16

**Authors:** Sara Mayo, Santiago Gutiérrez, Monica G. Malmierca, Alicia Lorenzana, M. Piedad Campelo, Rosa Hermosa, Pedro A. Casquero

**Affiliations:** ^1^Research Group of Engineering and Sustainable Agriculture, Natural Resources Institute, University of LeónLeón, Spain; ^2^Area of Microbiology, University School of Agricultural Engineers, University of LeónPonferrada, Spain; ^3^Department of Microbiology and Genetics, Spanish-Portuguese Centre for Agricultural Research, University of SalamancaSalamanca, Spain

**Keywords:** antifungal activity, defense-related genes, qPCR, ergosterol, squalene

## Abstract

Many *Trichoderma* species are well-known for their ability to promote plant growth and defense. We study how the interaction of bean plants with *R. solani* and/or *Trichoderma* affect the plants growth and the level of expression of defense-related genes. *Trichoderma* isolates were evaluated *in vitro* for their potential to antagonize *R. solani*. Bioassays were performed in climatic chambers and development of the plants was evaluated. The effect of *Trichoderma* treatment and/or *R. solani* infection on the expression of bean defense-related genes was analyzed by real-time PCR and the production of ergosterol and squalene was quantified. *In vitro* growth inhibition of *R. solani* was between 86 and 58%. In *in vivo* assays, the bean plants treated with *Trichoderma harzianum* T019 always had an increased size respect to control and the plants treated with this isolate did not decrease their size in presence of *R. solani*. The interaction of plants with *R. solani* and/or *Trichoderma* affects the level of expression of seven defense-related genes. Squalene and ergosterol production differences were found among the *Trichoderma* isolates, T019 showing the highest values for both compounds. *T. harzianum* T019 shows a positive effect on the level of resistance of bean plants to *R. solani*. This strain induces the expression of plant defense-related genes and produces a higher level of ergosterol, indicating its ability to grow at a higher rate in the soil, which would explain its positive effects on plant growth and defense in the presence of the pathogen.

## Introduction

The common bean (*Phaseolus vulgaris* L.) is the third most important food legume crop worldwide, surpassed only by the soybean [*Glycine max* (L.) Merr.] and peanut (*Arachis hypogea* L.). Among the southern countries of the European Union, Spain together with Italy and Greece are the main common bean producers. León, a province located at the northwest of Spain, is the main producer province by quantity and quality, with almost 45% of Spanish production in 2014. Socio-economic conditions of León province enabled possible the maintenance of local varieties in traditional cropping systems, which are based in small-scale farms (Casquero et al., 2006). The high quality of this legume has been awarded with a Protected Geographic Indication (PGI) (EC Reg. n.256/2010 published on 26 March 2010, OJEU L880/17). In the last few years, however dry bean production has gone through difficulties due to relatively low yields (mainly caused by fungus, virus, and bacteria) and insufficient income for growers.

Root rots are the main diseases caused by soil fungi having their incidence on bean yield. *Rhizoctonia solani* JG Kühn [Teleomorph: *Thanatephorus cucumeris* (AB Frank) Donk] is the main root rot in León, being detected in 91.8% of affected plants in an evaluation of its occurrence in bean plants (Valenciano et al., 2006). Plant infection occurs through wounds or by a coating of an organ with mycelium, which tears the cuticle and penetrates the epidermis. This pathogen is more aggressive at temperatures between 15 and 18°C and in moist soils. It is a necrotrophic pathogen, distributed worldwide (Guerrero-González et al., 2011). *R. solani* is one of the root and hypocotyl pathogen that causes most economic losses worldwide.

*Trichoderma* (Teleomorph: *Hypocrea*) is a fungal genus that is found in the soil. It is a secondary fast growing opportunistic invasive, which produces large numbers of spores, enzymes able to degrade the fungal cell wall (chitinases, glucanases, and proteases) and compounds with antimicrobial activity. Many *Trichoderma* species are also well known as biocontrol agents (BCA) of important phytopathogenic fungi. The primary mechanisms of biocontrol used by *Trichoderma* in direct confrontation with pathogenic fungi are the mycoparasitism (Papavizas, 1985) antibiosis, and competition for nutrients with the pathogen (Harman and Kubicek, 1998).

Many *Trichoderma* species colonize the root surface and cause substantial changes in plant metabolism (Harman et al., 2004). The physical interaction between *Trichoderma* and plants is limited to the first cell layer of the epidermis and the root bark. This symbiotic relationship would thus protect plants against pathogens. *Trichoderma* induces the expression of genes involved in defense response and promotes plant growth, root development and nutrient availability (Hermosa et al., 2012). During the *Trichoderma*-plant interaction various classes of metabolites could induce resistance such as proteins with enzymatic activity, low molecular weight compounds, related to the fungal or the plant cell wall, originated by the enzymatic activity of *Trichoderma* (Woo et al., 2006; Woo and Lorito, 2007) and other secondary metabolites that trigger plant defense mechanisms against the pathogen (Hermosa et al., 2012; Malmierca et al., 2014), by inducing the expression of pathogenesis–related (PR) proteins that reduce the diseases symptoms. Thus, when the plant contacts with a pathogen it is activated a mechanism of systemic acquired resistance (SAR). However, when they interact with a non-pathogen organism the plants activated a mechanism for induced systemic resistance (ISR) (Hermosa et al., 2013; Mukherjee et al., 2013).

Squalene is a polyunsaturated terpene that is an intermediate in the ergosterol biosynthetic pathway, which has an essential function in the fungal cell structure. The levels of squalene will influence the level of ergosterol biosynthesis (Garaiová et al., 2013). In addition to its structural function, and as a result of its importance in fungal development, ergosterol is also able to activate the expression of a number of defense genes and could increase the resistance of plants against pathogens (Lochman and Mikeš, 2005).

In this work 23 *Trichoderma* isolates were collected from bean fields. These isolates were used to study their effect on the growth of bean plants, and also in the defense response of plants against the phytopathogen *R. solani*. Thus, parameters as plant growth in the presence of the pathogen and/or the different *Trichoderma* isolates were evaluated, also analyzing the level of expression of defense-related genes in plants treated with the selected *Trichoderma* isolate.

## Materials and methods

### *Trichoderma* and *R. solani* isolates and culture collections

The present study was conducted with twenty-three isolates of *Trichoderma* (Table 1) collected from the production area of the Protected Geographical Indication (PGI), called “Alubia La Bañeza—León,” without any genetic manipulation and three isolates from other collections. The *Trichoderma* isolates were stored in the collection “Pathogens and Antagonists of the Laboratory Diagnosis of Pests and Diseases” (PALDPD, University of León, León, Spain). *R. solani* R43 was also collected from plants of the same PGI and selected by its high virulence (Table 1).

**Table 1:** *****Trichoderma*** and ***Rhizoctonia*** strains used in this study**.

**Lab. Code**	Culture collection^***^	**Localization/Received as**	**Identified as/References**
T001^*^	PAULET20	Bustillo del Páramo (León)	*T. harzianum*
T002^*^	PAULET21	Roperuelos del Páramo (León)	*Trichoderma* spp.
T003^*^	PAULET22	Bercianos del Páramo (León)	*Trichoderma* spp.
T004^*^	PAULET23	Riego de la Vega (León)	*T. gamsii*
T005^*^	PAULET24	Valderrey (León)	*T. longibrachiatum*
T006^*^	PAULET25	San Esteban de Nogales (León)	*Trichoderma* spp.
T007^*^	PAULET26	Villamejil (León)	*Trichoderma* spp.
T008^*^	PAULET27	Fresno de la Vega (León)	*T. citrinoviride*
T009	PAULET28	Bustillo del Páramo (León)	*Trichoderma* spp.
T010	PAULET29	Bustillo del Páramo (León)	*T. harzianum*
T011^*^	PAULET30	San Pedro de Bercianos (León)	*T. harzianum*
T012^*^	PAULET31	Quintana del Castillo (León)	*T. harzianum*
T013^*^	PAULET32	Quintana del Castillo (León)	*T. atroviride*
T014	PAULET33	Santa Marina del Rey (León)	*Trichoderma* spp.
T015^*^	PAULET34	San Cristobal de la Polantera (León)	*T. harzianum*
T016	PAULET35	Bustillo del Páramo (León)	*Trichoderma* spp.
T017	PAULET36	Bustillo del Páramo (León)	*Trichoderma* spp.
T018	PAULET37	Urdiales del Páramo (León)	*Trichoderma* spp.
T019^*^	PAULET38	Carrizo de la Ribera (León)	*T. harzianum*
T020	PAULET39	Soto de la Vega (León)	*T. harzianum*
T021	PAULET40	Pozuelo del Páramo (León)	*T. harzianum*
T022	PAULET41	Villaornate (León)	*Trichoderma* spp.
T023	PAULET42	Cabreros del Río (León)	*Trichoderma* spp.
T024^*^	IMI 352941	*T. atroviride*	Hermosa et al., 2000
T025^*^	NBT 59	*T. virens*	Hermosa et al., 2004
T34^**^	CECT 2413	*T. harzianum*	Kullnig et al., 2001
R43	PAULER006	Santa María del Páramo (León)	*Rhizoctonia solani*

* *Strains included in in vivo assays*.

** *Strain only used in ergosterol and squalene assays*.

*** *All PAULE strains are in Pathogens and Antagonists of the Laboratory Diagnosis of Pests and Diseases (PALDPD) Collection, University of León, León, Spain; IMI, CABI Bioscience (Egham); NBT, Newbiotechnic S.A. (Seville); CECT, Spanish Type Culture Collection, Burjassot, Spain*.

### *In vitro* antifungal assays

*Trichoderma* isolates were evaluated for their *in vitro* potential to antagonize the plant pathogenic fungus *R. solani* using two different tests. For all tests, plugs of 7 mm diameter collected from the edge of growing fungal colonies were used to inoculate potato dextrose agar medium (PDA) in sterile Petri dishes of 9 cm diameter. The dishes were incubated in the dark at 22°C for 7 days.

The aim of these tests was to study the percentage of *R. solani* growth inhibition caused by the different *Trichoderma* isolates.

**Figure 1:**
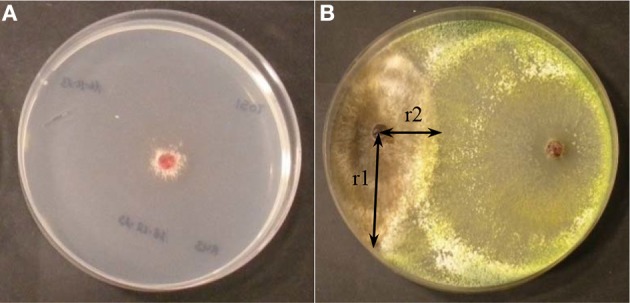
**(A)** Inhibition of *R. solani* growth by metabolites of Trichoderma spp in assay on membranes **(B)** Growth of Trichoderma (right) and *R. solani* (left) in direct confrontation assays. Parameters to calculate the percentage of inhibition of growth in direct confrontation (ID) % ID = [(r1−r2)/r1] x 100.

The antifungal assay on membranes was used to quantify the ability of the *Trichoderma* isolates to produce metabolites and/or enzymes with inhibitory activity against *R. solani*. The surface of Petri dishes containing PDA medium was overlaid with a sterile cellophane membrane. *Trichoderma* plugs, extracted from PDA dishes grown for 7 days at 22°C, were placed in the center of the dish with the cellophane sheet, containing PDA medium, and incubated for 48 h at 22°C. Then, the cellophane membranes along with the mycelia of *Trichoderma* isolates were removed and *R. solani* plugs were placed in the same plates. Growth of *R. solani* was recorded after 72 h to calculate the percentage of pathogen growth inhibition (Figure 1A). Control PDA plates of *R. solani*, where *Trichoderma* spp. had not been previously grown, were also prepared in the same conditions as above. The percentage of inhibition (IM) was calculated after 3 days of growth of *R. solani* in this medium using the formula %IM = [(C–T)/C]x100 (C: diameter of the *R. solani* control; T: diameter of *R. solani* after being exposed to the metabolites of *Trichoderma* spp.). Experiments were performed with four replicates. The results were compared by analysis of variance (ANOVA) and Fisher least significant difference (LSD) tests using SAS (SAS Institute Inc., 2004, Cary, NC, USA).

The direct confrontation assays were used to verify the ability of *Trichoderma* spp. to overgrow the pathogen. Each *Trichoderma* isolate was grown in dual culture with *R. solani* R43. The isolates were placed 5.5 cm apart on the same plate and incubated at 22°C for 5 days. Experiments were performed with four replicates. The parameters were measured after 5 days: r1 (distance between the pathogen sowing point and furthest point of the colony) and r2 (distance between the pathogen sowing point and the edge of the colony) from where *R. solani* and *Trichoderma* mycelia came into contact. Thus, the percentage of inhibition in the direct confrontation assay (ID) was calculated by the formula: %ID = [(r1–r2)/r1]x100 (Figure 1B). Inhibition of *R. solani* growth was compared by analysis of variance (ANOVA) and Fisher least significant difference (LSD) tests using SAS. (SAS Institute Inc., 2004, Cary, NC, USA).

### *In vivo* assay of the antifungal activity

These assays were only performed with those *Trichoderma* isolates that gave percentages of inhibition greater that 40% in membrane assays or 20% in direct confrontation assays, and that were able to sporulate on PDA medium.

The bioassays were performed in climatic chambers with 32 treatments as follow: 15 *in vitro* selected *Trichoderma* isolates against *R. solani* (R43) in order to test the antagonistic activity (RT0-number of *Trichoderma* isolate); 15 *in vitro* selected *Trichoderma* isolates in order to test their effect on plant (CT0-number of *Trichoderma* isolate); one control with *R. solani* (RC); and one control without fungi (CC). Thirty pots were used per treatment, with polypropylene pots (1 liter capacity) with substrate (80% white peat, 20% black peat and 5.5 pH). Each pot was watered with 250 ml of water prior inoculation. *R. solani* R43 was inoculated by surface irrigation with 50 ml per pot of a suspension of triturated micromicete culture of this pathogen using five Petri dishes (18 ml of PDA per dish) per liter of water. For control inoculation, only PDA medium was used without any pathogen. Pots were kept in a growth chamber for 8 days at 25°C (16 h) and 16°C (8 h), 60% relative humidity (RH) in the dark.

*Trichoderma* isolates were inoculated on PDA medium to grow in dark conditions (25°C) for 1 week. After that, they were exposed to light in order to induce the formation of spores. Spores suspensions were prepared at a final concentration of 2 × 10^7^ spores/ml. Bean seeds of “Canela” variety were surface sterilized (sodium hypochlorite 1% for 3 min and distilled water for 6 min). Then, they were coated with a spore suspension of each *Trichoderma* isolate. The seeds were submerged in the spore suspension (45 seeds per 20 ml spores suspension) and they were dried in a flow chamber for 12 h. Coated seeds were sown after 8 days of the inoculation of *R. solani* R43. The culture was maintained for 45 days with a photoperiod of 16 h light, 25°C/16°C (day/night), 60% RH and brightness of 3500 lux. Irrigations were performed every 4 days with tap water (about 250 ml/pot). On the 2nd–4th week a nutrient solution was added (Rigaud and Puppo, 1975). Plants were removed after 45 days from sowing, tissues with symptoms were placed in PDA medium, incubating the plates at 22°C for 5 days and identifying the fungus for fulfill Koch's postulates. The next parameters were evaluated in removed plants after 45 days from sowing: wet weight and dry weight (72 h in an oven, 82°C) of the aerial part and root system.

The data were transformed by the formula $\sqrt{\text{x}+0.5}$ and they were compared by analysis of variance (ANOVA) and Fisher least significant difference (LSD) tests using SAS (SAS Institute Inc., 2004, Cary, NC, USA).

### Nucleic acid extraction and manipulation

Genomic DNA from those isolates showing positive and negative phenotypic effects on bean plants, were extracted by growing the *Trichoderma* isolates in PDB medium (potato dextrose broth). Mycelia were then recovered by filtration, washed with 0.9% NaCl and dried on absorbent filter paper. The procedure for fungal genomic DNA isolation was performed as previously described (Cardoza et al., 2006).

### PCR amplification, sequencing and DNA analysis

The amplification of the ITS regions of the nuclear rDNA gene cluster and an approximately 0.56 kb fragment of the *tef1* (translation elongation factor 1-α) gene were carried out with the primer pairs ITS1/ITS4 and EF1-728F/EF1-LLErev, respectively, as described previously (Hermosa et al., 2004). The PCR products were purified from agarose gels using the NucleoSpin Extract II Kit (Macherey-Nagel, Düren, Germany), according to the manufacturer's protocol. PCR fragments were sequenced in an ABI 377 Prism Sequencer (Applied Biosystems, Foster City, CA).

The *Trichoderma* ITS and *tef1* sequences obtained in this work were analyzed using the online interactive key (available from http://www.isth.info/tools/blast/index.php) (Druzhinina et al., 2005).

### Analysis of expression of bean defense-related genes

Three bean leaves from 45 day-old plants of each treatment were randomly collected and stored at −80°C until use. Leaves were detached from plants inoculated with *Trichoderma* isolate showing positive phenotypic results in the *in vivo* test. Leaves were then reduced to a fine powder in a mortar under liquid nitrogen. Plant RNA isolation were performed as previously described (Malmierca et al., 2013).

cDNA were synthesized using 1 μg total RNA and a Reverse Transcription System with an Oligo(dT)_15_ as the primer (Promega, Madison, WT). cDNA were quantified using a Nanodrop 2000 (Thermo Scientific, Wilmington, DE) and used for further studies.

### Real time-PCR analysis

In order to analyze the effect of *Trichoderma* treatment and/or *R. solani* infection of bean plants, oligonucleotides corresponding to seven defense-related genes were designed based on their available sequences (Table 2). *PR1, PR2, PR3*, and *PR4*, which encode for pathogenesis related proteins related to the salicylate (SA) pathway; *CH5b, CH1* encoding for related to the jasmonate/ethylene pathway (JA/ET), and *PAL* involved in the phenylpropanoid pathway, were selected to be analyzed in the present study. α*-actin* (Upchurch and Ramirez, 2010; Guerrero-González et al., 2011) and *PvEF1*α (this work) encoding genes were used as reference (housekeeping genes) for comparative analysis. The qPCR reactions were carried out using Step One Plus™ (Applied Biosystems, Foster City, CA). The reactions were performed in a total volume of 20 μl: 10 μl Power SYBR® Green PCR Master Mix (Applied Biosystems, USA), 0.4 μl Forward Primer 10 μM, 0.4 μl Reverse Primer 10 μM, 5 μl cDNA, and H_2_O to 20 μl. The REST 2009^©^software (Pfaffl et al., 2002) was used to calculate the relative expression ratio and the significance of the differences between the gene expression levels. For each primer pair used in this work, we performed a standard curve with 320, 160, 80, 40, 20, and 10 ng cDNA to determine the PCR amplification efficiency (E value). Each measurement was made in triplicate (Malmierca et al., 2013).

**Table 2:** **Oligonucleotides designed for Real-Time PCR analysis**.

**Gene**	**Function gene**	**GenBank Accession number**	**Oligonucleotide name**	**Oligonucleotide sequence (5′-3′)**	**References**
*PvEF1α*	Elongation Factor 1	EF660340.1	EF1α-F	CGGGTATGCTGGTGACTTTT	This work
			EF1α-R	CACGCTTGAGATCCTTGACA	
*α-actin*	Actine	U60500.1	α-actin-F	GAGCTATGAATTGCCTGATGG	This work
			α-actin-R	CGTTTCATGAATTCCAGTAGC	
*PvCH5b*	Chitinase	FE897014.1	CH5b-F	CAGCCAAAGGCTTCTACACC	This work
			CH5b-R	TTGTTTCGTGAGACGTTTGC	
*PvPR1*	Pathogenesis related 1	HO864272.1	PR1-F	TGGTCCTAACGGAGGATCAC	This work
			PR1-R	TGGCTTTTCCAGCTTTGAGT	
*PvPR2*	Pathogenesis related 2	HO864270.1	PR2-F	GTGAAGGACGCCGATAACAT	This work
			PR2-R	ACTGAGTTTGGGGTCGATTG	
*PvPR4*	Pathogenesis related 4	HO864354.1	PR4-F	CGCAGTGAGTGCATATTGCT	This work
			PR4-R	TGTTTGTCACCCTCAAGCAC	
*PvCH1*	Chitinase				Pereira et al., 2014
*PvPR3*	Pathogenesis related 3				Pereira et al., 2014
*PvPAL*	Phenylalanine ammonia-lyase				Pereira et al., 2014

### Quantification of ergosterol and squalene

The *Trichoderma* selected strain was inoculated in 100 ml of CM medium (0.5% malt extract, 0.5% yeast extract, and 0.5% glucose) with 10^6^ spores/ml, and incubated 24 h at 28°C. Then 20 ml from the previous cultures were inoculated on 100 ml potato dextrose broth (PDB medium) and were incubated as before during 24–96 h. The mycelia were filtered through nytal filters (30 μm diameter) and the liquid removed by drying between filter papers. The dry weight of the fungal pellet was calculated. Total intracellular sterols were extracted and ergosterol and squalene content were quantified as previously reported other authors (Cardoza et al., 2007; Ghimire et al., 2009). All measurements were made in duplicate in the *Trichoderma* selected isolate and a strain used as control of the same species of the selected isolate. The results were compared by analysis of variance (ANOVA) and Fisher least significant difference (LSD) tests using SAS (SAS Institute Inc., 2004, Cary, NC, USA).

## Results

### Analysis of the *in vitro* antagonistic activity of *Trichoderma* isolates with *R. solani*

The first test to determine the *in vitro* antifungal ability of the different *Trichoderma* isolates was based on their ability to produce metabolites that may inhibit the growth of *R. solani* (Table 3, Figure 1A). *Trichoderma* isolates T003, T004, T006, T020, T022, T012, T013, T025, T016, T007, T024, T005, and T010 inhibited *R. solani* growth by more than 75%, with the highest inhibition produced by T003, T004, T006, T020, and T022 (86.70%). T019, T008, T002, T021, T001, T018, and T023 showed a remarkable inhibition (75–40%). Finally, T015, T014, T017, T011, and T009 inhibited *R. solani* growth by less than 40%, and T009 showed the lowest percentage (15.82%).

**Table 3:** *****In vitro*** antifungal activity of ***Trichoderma*** strains against ***R. solani*** R43**.

**Strain**	**Inhibition in growth assay on membranes (% ± Standard error) (1)**		**Strain**	**Inhibition in growth assay on direct confrontation (% ± Standard error) (2)**	
T003^*^	86.70 ± 0.15	a	T021	72.77 ± 4.49	a
T004^*^	86.70 ± 0.15	a	T004^*^	47.13 ± 0.91	b
T006^*^	86.70 ± 0.15	a	T013^*^	43.49 ± 1.41	bc
T020	86.70 ± 0.15	a	T011^*^	42.57 ± 2.00	bcd
T022	86.70 ± 0.15	a	T014	41.99 ± 1.33	cde
T012^*^	86.67 ± 0.14	a	T023	41.57 ± 1.40	cdef
T013^*^	85.04 ± 1.04	a	T018	41.01 ± 1.93	cdefg
T025^*^	83.97 ± 1.97	a	T020	39.21 ± 1.41	cdefgh
T016	82.29 ± 2.33	a	T006^^*^^	38.68 ± 0.59	defghi
T007^*^	76.77 ± 2.52	b	T017	37.42 ± 1.61	efghij
T024^*^	76.22 ± 2.32	bc	T016	37.10 ± 0.63	fghij
T005^*^	75.65 ± 2.23	bc	T002^^*^^	36.44 ± 0.29	ghij
T010	75.14 ± 3.30	bc	T024^^*^^	36.43 ± 3.71	hij
T019^*^	72.36 ± 2.01	bcd	T007^*^	36.34 ± 1.30	hij
T008^*^	70.61 ± 1.82	cde	T005^*^	36.16 ± 0.67	hij
T002^*^	66.75 ± 1.18	def	T019^*^	35.09 ± 1.01	hijk
T021	65.98 ± 0.77	ef	T022	35.05 ± 0.34	hijk
T001^*^	65.51 ± 1.75	f	T008^*^	35.04 ± 0.89	hijk
T018	54.12 ± 2.35	g	T012^*^	34.59 ± 0.98	ijk
T023	48.26 ± 3.10	h	T025^*^	33.28 ± 4.24	kjl
T015^*^	39.99 ± 0.82	i	T010	30.55 ± 1.32	kl
T014	38.82 ± 2.96	ij	T015^*^	28.84 ± 0.85	lm
T017	34.10 ± 0.88	j	T001^*^	24.66 ± 0.74	m
T011^*^	21.40 ± 2.66	k	T003^^*^^	19.85 ± 2.97	n
T009	15.82 ± 1.40	l	T009	14.63 ± 0.83	o

*Values in the same column followed by different letters are significantly different (Fisher's LSD. p < 0.05)*.

*(1) Growth assay on membranes (effect of Trichoderma isolates on percent growth inhibition of R. solani. using cellophane membranes. (2) in direct confrontation assays (percentage of inhibition of R43 growth when grown confronted with Trichoderma strains during 5 days in PDA medium plates)*.

* *Trichoderma isolates selected for in vivo experiments because they were able to sporulate in PDA medium and showed a percentage of inhibition higher than 40% in the membrane assays. and/or 20% in the direct confrontation assays*.

T021 was the *Trichoderma* isolate showing the highest percentage of inhibition (72.77%) in the direct confrontation assays (Table 3, Figure 1B), whereas T009 showed the lowest inhibition values (14.63%). The inhibition percentages detected for the other *Trichoderma* isolates ranged from 47.13 to 30.55%.

### Analysis of the *in vivo* antagonistic activity of *Trichoderma* isolates with *R. solani*

The results of the *in vitro* membrane assays and direct confrontation assays against *R. solani*, showed above, were used to select the isolates that would be used for the *in vivo* analysis. Thus, fifteen *Trichoderma* isolates (marked with asterisk in Tables 1, 3) were tested since they were able to sporulate in PDA medium and showed a percentage of inhibition of *R. solani* growth higher than 40% in the membrane assays, and/or 20% in the direct confrontation assays.

On plants removed after 45 days from sowing for Koch‘s postulates, *R. solani* was found and *Trichoderma* isolates were also present in the medium.

**Figure 2:**
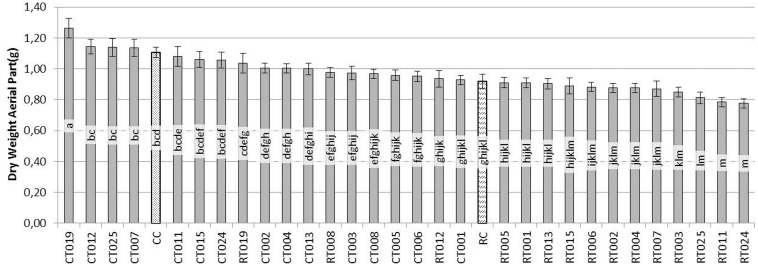
**Evaluation of the dry weight (g) of the aerial parts of bean plants grown during 45 days after sowing with 60 replicates**. [*Trichoderma* isolates without pathogen (CT0-number of *Trichoderma* isolate), *Trichoderma* isolates with *R. solani* (RT0-number of *Trichoderma* isolate), *R. solani* control (RC) and control without fungus (CC)].

When dry aerial parts (Figure 2) were analyzed, plants treated with CT019 had the greatest weight of the aerial part, being significantly different from the control (CC). In the RT019 treatment, plants did not show significant differences in comparison with the control (CC), what is indicative of a biocontrol effect by T019 isolate.

**Figure 3:**
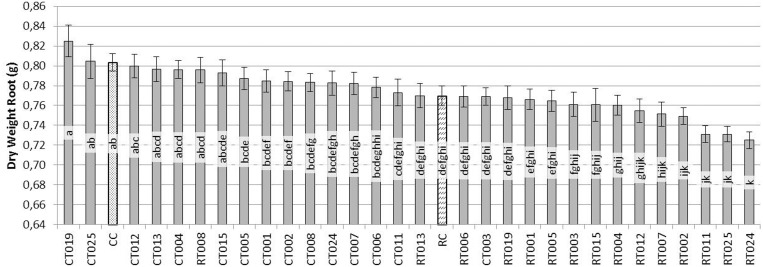
**Evaluation of the dry weight (g) of the root system of bean plants grown for 45 days after sowing with 60 replicates**. [*Trichoderma* isolates without pathogen (CT0-number of *Trichoderma* isolate), *Trichoderma* isolates with *R. solani* (RT0-number of *Trichoderma* isolate), *R. solani* control (RC) and control without fungus (CC)].

In the case of the root system (Figure 3), the situation was similar to that observed in the aerial parts. CT019 treated plants were not significantly different in dry weight, although it was always greater than the weight of the control plants (CC). If the pathogen was present in the soil with same *Trichoderma* isolate, RT019 treatment, in the case of wet weight, there were no significant differences between control (CC) and control pathogen (RC) (data not shown).

**Figure 4:**
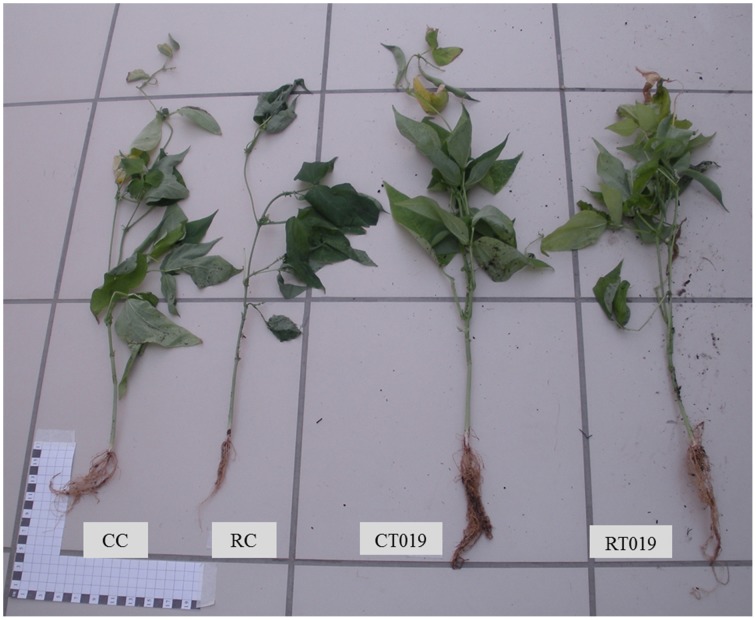
**Differences in growth of bean plants**. CC plant control; RC plant with *R. solani*, CT019 plant with *Trichoderma* T019; RT019 plant with *R. solani* and *Trichoderma* T019.

Based on these results, the *Trichoderma* isolate T019 was selected for further studies since it showed the best positive effects on plant phenotype among all the analyzed isolates (Figure 4).

### Molecular identification of *Trichoderma* isolates

Those *Trichoderma* isolates able to sporulate in PDA medium, showing a percentage of inhibition higher than 40% in the membrane assays, and/or 20% in the direct confrontation assays, were identified. ITS1 region of rDNA and a fragment of the translation elongation factor 1 (*tef1*) were amplified and sequenced for 12 isolates, and both nucleotide sequences were used for identification at species level. Eight out of these 12 isolates were identified as *T. harzianum*, and *T. atroviride, T. gamsii, T. longibrachiatum* and *T. citrinoviride* species were represented by one isolate (Table 1).

### Effect of *Trichoderma* treatment and/or *R. solani* infection in the expression of bean defense related genes

The amplification efficiencies of the oligo-pairs (Table 2) were: α*-actin* 1.150, *PvEF1*α 0.903, *CH5b* 0.883, *CH1* 1.098, *PR1* 1.094, *PR2* 1.048, *PR3* 0.947, *PR4* 0.922, and *PAL* 0.962.

**Figure 5:**
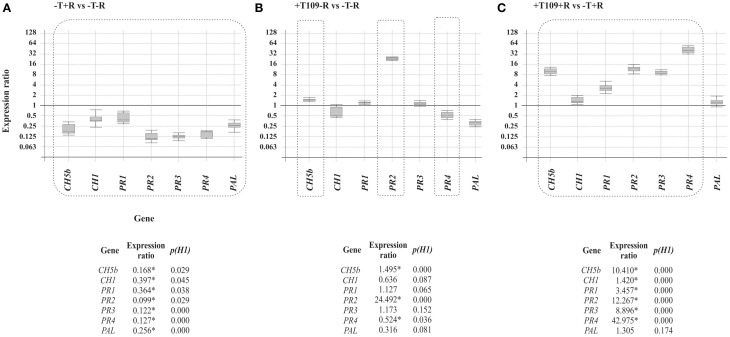
**Expression of ***CH5b, CH1, PR1, PR2**, **PR3, PR4***, and ***PAL*** genes in comparison with α***-actin*** and ***PvEF-1***α reference genes**. The comparisons and statistical analysis were performed using the REST2009^©^program (Pfaffl et al., 2002). The numeric data are illustrated at the bottom of the figure and those showing differences statistically significant (*p* < 0.05) are indicated with an asterisk and they are boxed in the graphic representation. Comparison of the gene expression of the bean defense-related genes **(A)** in plants infected with *R. solani* R43 vs. control plants. **(B)** In plants treated with *Trichoderma* T019 vs. control plants **(C)** In plants infected with *R. solani* R43 and treated with *Trichoderma* T019 vs. plants infected with *R. solani* R43.

α*-actin* and *PvEF1*α were used as housekeeping genes to determine the relative expression level of the other genes analyzed in the present work. *Trichoderma* T019 strain was selected, based on its positive effects on bean phenotype with and without *R. solani* infection. The results included in Figure 5 showed that: (i) *R. solani* down-regulated the expression of all the *P. vulgaris* defense-related genes analyzed (Figure 5A), raising values of expression ranging between 0.099 (*p* = 0.029) for *PR2* and 0.397 (*p* = 0.045) for *CH1*; (ii) In plants treated with T019 compared with control plants (bean plants not treated with *Trichoderma* nor infected with *R. solani*) only the *CH5b* and *PR2* were significantly up-regulated, raising comparative expression values of 1.495 (*p* = 0.000) and 24.492 (*p* = 0.000), respectively (Figure 5B). Finally, (iii) treatment with T019 and infection with *R. solani* significantly up-regulated the expression ratio of the analyzed genes, except *PAL*, in comparison with plants only infected with *R. solani*, with values ranging from 1.420 (*p* = 0.000) to 42.975 (*p* = 0.000) for *CH1* and *PR4* respectively (Figure 5C).

A non-remarkable significant up-regulation of the defense-related gene expression was observed when expression ratios of the selected genes in plants treated with *T. harzianum* T019 and infected with *R. solani* were compared with those from plants untreated or uninfected (data not shown).

### Production of ergosterol and squalene by the *Trichoderma* selected strains

Strain T019 (*T. harzianum*) was selected for this analysis because it was those producing the most remarkable positive phenotypic effect on beans (see above). *T. harzianum* T34 (Table 1) was used as control strain for comparative purposes because it is a well-known strain, widely characterized (Kullnig et al., 2001).

Production of ergosterol at 24 h of growth did not show significant differences between the strains *T. harzianum* (T34 and T019) (Table 4). When the production of ergosterol was analyzed at 96 h, T019 produced significantly higher amounts than its respective control (T34).

**Table 4:** **Ergosterol and squalene quantification from 24 and 96 h samples of the selected ***Trichoderma*** strains selected for this study**.

***Trichoderma* spp**.	**Ergosterol (mg E/g d wt)**		**Squalene (mg S/g d wt)**	
	**24 h**	**96 h**	**24 h**	**96 h**
T34	16.197 ± 1.019 a	8.970 ± 0.388 b	0.317 ± 0.020 a	0.052 ± 0.049 b
T019	14.336 ± 2.459 a	12.502 ± 3.568 a	0.303 ± 0.041 a	0.348 ± 0.103 a

*Values in the same column followed by different letters are significantly different (Fisher's LSD, p < 0.05)*.

Regarding the squalene production at 24 h, *T. harzianum* (T34 and T019) strains did not show significant differences. However, at 96 h of growth, T019 produced amounts significantly higher than its control.

## Discussion

Most of the *Trichoderma* isolates studied in the present work have been able to control the growth of *R. solani*. The percentage of growth inhibition in the direct confrontation assays raised values up to 72.77% for T021, but most of the isolates were between 47 and 24%. By contrast, in the antifungal assays on membranes, those percentages ranged mostly between 86 and 58%. In the assays conducted by Campelo et al. (2010) the percentage of inhibition in membrane assays using *T. virens* T59 (NBT59) and *T. atroviride* T11 (IMI352941) range between 100 and 84.7%, respectively. The differences observed in the different *in vitro* assays might be due to the variability of genotypes, with differences in growth, sporulation and in their environmental biological interactions as a consequence of the prevalence of different modes of action on each of the analyzed strains (Ruano-Rosa et al., 2010).

The *in vitro* antagonistic activity of the *Trichoderma* isolates is an indication of their *in vivo* biological activity against phytopathogenic fungi. However, it has been found that the *in vitro* antifungal activity of *Trichoderma* does not correlate in a direct way with its *in vivo* ability to control diseases caused by phytopathogenic fungi, since many other factors influence that activity (Anees et al., 2010).

Regarding the *in vivo* assays, bean plants that had been in contact with some *Trichoderma* isolate had an increased size when the pathogen was not present. Pereira et al. (2014) also observed that *T. harzianum* was able to promote the growth of common bean plants in comparison to plants grown in its absence. However, in the present work only T019 treated plants did not decrease their size in the presence of *R. solani*. These data are in agreement with those from Yedidia and coworkers reporting a much stronger effect on cucumber plants treated with *T. harzianum*, which increased by 75% the length of the root, 95% aerial parts, 80% dry weight and 80% the size of the blade relative to the untreated control (Yedidia et al., 2001). *T. harzianum* was able to promote the growth of common bean plants in comparison to plants grown in its absence (Pereira et al., 2014).

However, in the present work opposite results were observed to those previously reported (Tello et al., 1985), indicating that infection with *R. solani* not always resulted in a reduction of the bean plant size, which was explained as a result of the activation of the natural plant-defense mechanisms (Cardoso and Echandi, 1987) that would lead to an enhanced development of the plants when this pathogen was present in the soil. In the present study we have observed that treatment with *R. solani* resulted in a down-regulation of all the bean defense-related genes analyzed, indicating a certain compensation effect between the intensity of plant defense response and plant growth (Hermosa et al., 2013).

Little is known about the effect of *Trichoderma* treatment and/or infection with *R. solani* in the expression of bean defense-related gene expression. As indicated above, interaction of the plant with *R. solani* during 45 days of growth caused the repression of the seven defense-related genes studied (*CH5b, CH1, PR1, PR2, PR3, PR4*, and *PAL1*), as a mechanism to overcome the plant defense response and thus facilitating the progression of the infection process within the plant. Other authors have observed similar results in assays with tomato plants infected with *Pseudomonas syringae* pv. *tomato*, where a repression of *PR1* and *PR4* was shown, as an indication of the decrease in the plant self-defense mechanism, then facilitating disease progression (Zhao et al., 2003).

Expression of *P. vulgaris* defense-related genes was analyzed in leaves, even when the interaction with the pathogen *R. solani* is initially produced at the root level, to determine if the signals generated in roots as result of this interaction are able to systematically stimulate the bean defense along long distance from seed to leaf. When the interaction of plant with T019 was analyzed, *PR2* was up-regulated, *PR4* was slightly restrained but *PR1* and *PR3* were not affected. Pereira and coworkers observed that *T. harzianum* also seems to potentiate common bean response against the phytopathogenic fungus *R. solani*, as shown by the increase in the levels of *glu1* and *pod3* in the double treatment in comparison to that obtained for plants in the presence of *R. solani* alone (Pereira et al., 2014). Other studies showed that there was an increase in expression *PR1* at 16 h of interaction (Guerrero-González et al., 2011). Rivière and coworkers suggested that *PR1* was down-regulated by the β-1,3-glucanases (PR2 and PR3) (Rivière et al., 2008). In the present study, after 45 days of the inoculation of the biocontrol isolate we observed a higher expression of *PR2*, which could be due to an alteration of plant defense responses at these longer times in comparison with shorter ones assayed in the previous reported works. This would result in a higher β-1,3-glucanase activity in the cell wall that would increase oligosaccharides released by its action, then acting as elicitors of the plant defense response and/or of the fungal secondary metabolism (Druzhinina et al., 2011).

In the case of plants inoculated with *Trichoderma* and seeded in soil infected with *R. solani*, when compared with plants only infected with *R. solani*, thus excluding the effect due exclusively to this pathogen, a significant up-regulation of all the analyzed genes was observed, except *PAL*, indicating that in presence of *R. solani* several mechanisms are induced in *Trichoderma* that potentiate its ability to elicit plant defense-responses. In previous works, during the interaction of *Solanum tuberosum* with *R. solani* it was observed that after 120 h from the initial infection and damage to the first floor, new outbreaks produce less symptoms, suggesting that the plant might be prepared to defend and avoid disease progression (Lehtonen et al., 2008). Other studies have shown that the *PR4* was overexpressed in eggplants treated with BCAs suggesting that these agents promoted defensive reactions within the plant (Angelopoulou et al., 2014). In other assays with pepper plants an increase in the accumulation of PR1 and chitinases was also observed when these plants were previously inoculated with a strain of *Fusarium oxysporum* and subsequently exposed to *Verticillium dahliae* (Veloso and Díaz, 2012), emphasizing their importance in the response to pathogen infection and to abiotic stresses (Jung and Hwang, 2000). Thus, it could be inferred that the effect of *Trichoderma* would therefore modulate the response of the plant and prevent the suppression of defense genes caused by *R. solani*. However, the effect of the different *Trichoderma* strains in the different plants would indicate the existence of great differences between them.

In the case of *CH5b*, related to JA pathway, when *R. solani* was in contact with the plant for 45 days of development, the expression of this gene was down-regulated in comparison with non-infected plants. When bean plants were inoculated with *Trichoderma*, its expression was slightly induced. However, if the antagonist and the pathogen were present in the medium, the effect caused by *R. solani* was the overexpression of this gene. In previous works (Broglie et al., 1991) it was shown that *CH5b*, responsible for chitinase production, was overexpressed in *Nicotiana tabacum* and *Brassica napus* infected with *R. solani*, and their disease symptoms were reduced. Benhamou et al. (1993) found that the expression of this gene caused a reduction of disease in *B. napus* infected with the same pathogen, which correlates with similar results observed in strawberry plants inoculated with *Botrytis cinerea* (Vellicce et al., 2006). In the present study we observed that the contact of the plant with the pathogen during a period of 45 days resulted in a significant repression of this gene, similarly to what it was observed for the expression of *CH1*, which thereby facilitates disease progression. However, in the presence of *Trichoderma* a similar effect to that previously described for SA-related genes was observed, so facilitating a prevention of disease progression.

In the case of the phenylalanine ammonia lyase (PAL), this is a key enzyme in the metabolic pathway of phenylpropanoid compounds by catalyzing the amino acid L-phenylalanine deamination, giving rise to trans-cinnamic acid and ammonia. The trans-cinnamic acid is used for the synthesis of various phenolic compounds, which are precursors in the synthesis of esters, coumarins, flavonoids, and lignin. The production of this enzyme is controlled during plant growth, but is also induced in cells neighboring the infection site and, besides infection, various environmental stimuli such as injury, heavy metal contamination, light and growth regulators (Rahman and Punja, 2005). In the present work, we observed that this gene was down-regulated or not affected in all the conditions assayed, indicating that in the analyzed strain, PAL activity would be more focused in the plant response against environmental stress than in the response against pathogens, which contrast with the role of this enzyme described in pepper, where it is involved in the positive regulation of SA-dependent defense signaling (Kim and Hwang, 2014).

Regarding squalene and ergosterol production, in this study it was found that there were differences among the isolates. Ergosterol is a sterol found in the fungal membrane, which although considered by the plant as a PAMP (pathogen associated molecular patterns) (Nürnberger et al., 2004) triggers a series of reactions (Cervone et al., 1997) which would cause an activation of the genes of plant defense (Rossard et al., 2010). Squalene is a precursor of the ergosterol biosynthetic pathway (Malmierca et al., 2013) located in the cellular membranes or accumulated as droplets in the cytoplasm whose physiological function, apart from acting as an ergosterol precursor, still remains unclear, but it might play some role in the elicitation of plant defense responses. Thus, in this assay, an increased production of these compounds by *Trichoderma* would result in the induction of defense genes in the bean, then the plant could grow better under a pathogen presence in the soil.

## Conclusions

*Trichoderma* isolates inhibit the development of *R. solani* under *in vitro* conditions, when they grow in Petri dishes. *In vivo* conditions, the bean plants that had been in contact with the *Trichoderma* isolates always had an increased size when the pathogen was not present. When *R. solani* was present in the soil, the development of the bean plants was significantly reduced. However, bean plants treated with strain T019 did not decrease their size in the presence of *R. solani*.

The interaction of bean plants with *R. solani* caused, after 45 days of growth, the down-regulation of the seven defense-related genes studied (*CH5b, CH1, PR1, PR2, PR3, PR4, PAL*) as a mechanism to overcome the plant defense response and thus facilitating the progression of the infection process within the plant.

An increased production of ergosterol and squalene by *Trichoderma* resulted in the induction of defense genes in the bean plants. In this way, the plant would grow better under a pathogen presence in the soil.

*T. harzianum* T019 shows a positive effect on the level of resistance of bean plants to *R. solani*. This strain induces the expression of plant defense-related genes and produces a higher level of ergosterol, indicating its ability to grow at a higher rate in the soil, which would explain its positive effects on plant growth and defense in the presence of the pathogen.

### Conflict of interest statement

The authors declare that the research was conducted in the absence of any commercial or financial relationships that could be construed as a potential conflict of interest.

## References

[B1] Anees M. Tronsmo A. Edel-Hermann V. Hjeljord L. G. Héraud C. Steinberg C. (2010). Characterization of field isolates of *Trichoderma* antagonistic against *Rhizoctonia solani*. Fungal Biol. 114, 691–701. 10.1016/j.funbio.2010.05.007 20943179

[B2] Angelopoulou D. J. Naska E. J. Paplomatas E. J. Tjamos S. E. (2014). Biological control agents (BCAs) of verticillium wilt: influence of application rates and delivery method on plant protection, triggering of host defence mechanisms and rhizosphere populations of BCAs. Plant Pathol. 63, 1062–1069. 10.1111/ppa.12198

[B3] Benhamou N. Broglie K. Chet I. Broglie R. (1993). Cytology of infection of 35S-bean chitinase transgenic canola plants by *Rhizoctonia solan*i: cytochemical aspects of chitin breakdown *in vivo*. Plant J. 4, 295–305. 10.1046/j.1365-313X.1993.04020295.x

[B4] Broglie K. Chet I. Holliday M. Cressman R. Biddle P. Knowlton S.. (1991). Transgenic plants with enhanced resistance to the fungal pathogen *Rhizoctonia solani*. Science 254, 1194–1197. 17776411 10.1126/science.254.5035.1194

[B5] Campelo P. Cardoza R. Lorenzana A. Hermosa M. Monte E. Reinoso B. (2010). Biological control of phytopathogenic fungi in bean (*Phaseolus vulgaris* L.) with *Trichoderma atroviride* and *Trichoderma virens*. [Abstract]. Bean Improv. Coop. Colo. 53, 114–115.

[B6] Cardoso J. Echandi E. (1987). Biological control of Rhizoctonia root rot of snap bean with binucleate *Rhizoctonia*-like fungi. Plant Dis. 71, 167–170. 10.1094/PD-71-0167

[B7] Cardoza R. E. Hermosa M. R. Vizcaíno J. A. González F. Llobell A. Monte E.. (2007). Partial silencing of a hydroxy-methylglutaryl-CoA reductase-encoding gene in *Trichoderma harzianum* CECT 2413 results in a lower level of resistance to lovastatin and lower antifungal activity. Fungal Genet. Biol. 44, 269–283. 10.1016/j.fgb.2006.11.013 17218128

[B8] Cardoza R. E. Vizcaíno J. A. Hermosa M. R. Sousa S. González F. J. Llobell A.. (2006). Cloning and characterization of the erg1 gene of *Trichoderma harzianum*: effect of the erg1 silencing on ergosterol biosynthesis and resistance to terbinafine. Fungal Genet. Biol. 43, 164–178. 10.1016/j.frb.2005.11.002 16466954

[B9] Casquero P. A. Lema M. Santalla M. De Ron A. M. (2006). Performance of common bean (*Phaseolus vulgaris* L.) landraces from Spain in the Atlantic and Mediterranean environments. Genet. Res. Crop Evol. 53, 1021–1032. 10.1007/s10722-004-7794-1

[B10] Cervone F. Castoria R. Leckie F. De Lorenzo G. (1997). Perception of fungal elicitors and signal transduction, in Signal Transduction in Plants, ed Aducci P. (Basel, Boston, Berlin: Birkhäuser), 153–177.

[B11] Druzhinina I. S. Kopchinskiy A. G. Komon M. Bissett J. Szakacs G. Kubicek C. P. (2005). An oligonucleotide barcode for species identification in *Trichoderma* and *Hypocrea*. Fungal Genet. Biol. 42, 813–828. 10.1016/j.fgb.2005.06.007 16154784

[B12] Druzhinina I. S. Seidl-Seiboth V. Herrera-Estrella A. Horwitz B. A. Kenerley C. M. Monte E.. (2011). *Trichoderma*: the genomics of opportunistic success. Nat. Rev. Microbiol. 9, 749–759. 10.1038/nrmicro2637 21921934

[B13] Garaiová M. Zambojová V. Šimová Z. Griac P. Hapala I. (2013). Squalene epoxidase as a target for manipulation of squalene levels in the yeast *Saccharomyces cerevisiae*. FEMS Yeast Res. 14, 310–323. 10.1111/1567-1364.12107 24119181

[B14] Ghimire G. P. Hei C. L. Jae K. S. (2009). Improved squalene production via modulation of the methylerythritol 4-phosphate pathway and heterologous expression of genes from *Streptomyces peucetius* ATCC 27952 in *Escherichia coli*. Appl. Environ. Microbiol. 75, 7291–7293. 10.1128/AEM.01402-09 19767465 PMC2786506

[B15] Guerrero-González M. L. Rodríguez-Kessler M. Rodríguez-Guerra R. González-Chavira M. Simpson J. Sanchez F.. (2011). Differential expression of *Phaseolus vulgaris* genes induced during the interaction with *Rhizoctonia solani*. Plant Cell Rep. 30, 1465–1473. 10.1007/s00299-011-1055-5 21416283

[B16] Harman G. E. Howell C. R. Viterbo A. Chet I. Lorito M. (2004). *Trichoderma* species–opportunistic, avirulent plant symbionts. Nat. Rev. Microbiol. 2, 43–56. 10.1038/nrmicro797 15035008

[B17] Harman G. E. Kubicek C. P. (1998). Trichoderma and Gliocladium: Enzymes, Biological Control and Commercial Applications. Vol. 2, London: CRC Press.

[B18] Hermosa M. R. Grondona I. Iturriaga E. A. Díaz-Mínguez J. M. Castro C. Monte E.. (2000). Molecular characterization and identification of biocontrol isolates of *Trichoderma* spp. Appl. Environ. Microbiol. 66, 1890–1898. 10.1128/AEM.66.5.1890-1898.2000 10788356 PMC101429

[B19] Hermosa M. R. Keck E. Chamorro I. Rubio B. Sanz L. Vizcaíno J. A.. (2004). Genetic diversity shown in *Trichoderma* biocontrol isolates. Mycol. Res. 108, 897–906. 10.1017/S0953756204000358 15449594

[B20] Hermosa R. Belén Rubio M. Cardoza R. E. Nicolás C. Monte E. Gutiérrez S. (2013). The contribution of *Trichoderma* to balancing the costs of plant growth and defense. Int. Microbiol. 16, 69–80. 10.2436/20.1501.01.181 24400524

[B21] Hermosa R. Viterbo A. Chet I. Monte E. (2012). Plant-beneficial effects of *Trichoderma* and of its genes. Microbiol 158, 17–25. 10.1099/mic.0.052274-0 21998166

[B22] Jung H. W. Hwang B. K. (2000). Pepper gene encoding a basic ß-1,3-glucanase is differentially expressed in pepper tissues upon pathogen infection and ethephon or methyl jasmonate treatment. Plant Sci. 159, 97–106. 10.1016/S0168-9452(00)00334-4 11011097

[B23] Kim D. S. Hwang B. K. (2014). An important role of the pepper phenylalanine ammonia-lyase gene (PAL1) in salicylic acid-dependent signalling of the defence response to microbial pathogens. J. Exp. Bot. 65, 2295–2306. 10.1093/jxb/eru109 24642849 PMC4036500

[B24] Kullnig C. Krupica T. Woo S. L. Mach R. L. Rey M. Benítez T. (2001). Confusion abounds over identities of *Trichoderma* biocontrol isolates. Mycol. Res. 105, 770–772. 10.1017/S0953756201229967

[B25] Lehtonen M. J. Somervuo P. Valkonen J. P. T. (2008). Infection with *Rhizoctonia solan*i induces defense genes and systemic resistance in potato sprouts grown without light. Phytopathol 98, 1190–1198. 10.1094/PHYTO-98-11-1190 18943407

[B26] Lochman J. Mikeš V. (2005). Activation of different defence-related genes expression by ergosterol. FEBS J. 272, 470. 10.1111/j.1742-4658.2005.4739_12.x15988564

[B27] Malmierca M. G. Barua J. Mccormick S. P. Izquierdo-Bueno I. Cardoza R. E. Alexander N. J.. (2014). Novel aspinolide production by *Trichoderma arundinaceum* with a potential role in *Botrytis cinerea* antagonistic activity and plant defence priming. Environ. Microbiol. 17, 1103–1118. 10.1111/1462-2920.12514 24889745

[B28] Malmierca M. G. Cardoza R. E. Alexander N. J. McCormick S. P. Collado I. G. Hermosa R.. (2013). Relevance of trichothecenes in fungal physiology: disruption of tri5 in *Trichoderma arundinaceum*. Fungal Genet. Biol. 53, 22–33. 10.1016/j.fgb.2013.02.001 23454546

[B29] Mukherjee P. K. Horwitz B. A. Herrera-Estrella A. Schmoll M. Kenerley C. M. (2013). *Trichoderma* research in the genome era. Annu. Rev. Phytopathol. 51, 105–129. 10.1146/annurev-phyto-082712-102353 23915132

[B30] Nürnberger T. Brunner F. Kemmerling B. Piater L. (2004). Innate immunity in plants and animals: Striking similarities and obvious differences. Immunol. Rev. 198, 249–266. 10.1111/j.0105-2896.2004.0119.x 15199967

[B31] Papavizas G. (1985). *Trichoderma* and *Gliocladium*: biology, ecology, and potential for biocontrol. Annu. Rev. Phytopathol. 23, 23–54. 10.1146/annurev.py.23.090185.000323

[B32] Pereira J. L. Queiroz R. M. L. Charneaum S. O. Felix C. R. Ricart C. A. O. Lopes Da Silva F.. (2014). Analysis o*f Phaseolus vulgaris* response to its association with *Trichoderma harzianum* (ALL-42) in the presence or absence of the phytopathogenic fungi *Rhizoctonia solani* and *Fusarium solani*. PLoS ONE 9:e98234. 10.1371/journal.pone.0098234 24878929 PMC4039509

[B33] Pfaffl M. W. Horgan G. W. Dempfle L. (2002). Relative expression software tool (REST) for group-wise comparison and statistical analysis of relative expression results in real-time PCR. Nucleic Acids Res. 30:e36. 10.1093/nar/30.9.e36 11972351 PMC113859

[B34] Rahman M. Punja Z. K. (2005). Biochemistry of ginseng root tissues affected by rusty root symptoms. Plant Physiol. Biochem. 43, 1103–1114. 10.1016/j.plaphy.2005.09.004 16386432

[B35] Rigaud J. R. Puppo A. (1975). Indole 3 acetic acid catabolism by soybean bacteroids. J. Gen. Microbiol. 88, 223–228. 10.1099/00221287-88-2-223

[B36] Rivière M. Marais A. Ponchet M. Willats W. Galiana E. (2008). Silencing of acidic pathogenesis-related PR-1 genes increases extracellular β-(1 → 3)-glucanase activity at the onset of tobacco defence reactions. J. Exp. Bot. 59, 1225–1239. 10.1093/jxb/ern044 18390849

[B37] Rossard S. Roblin G. Atanassova R. (2010). Ergosterol triggers characteristic elicitation steps in *Beta vulgaris* leaf tissues. J. Exp. Bot. 61, 1807–1816. 10.1093/jxb/erq047 20304987

[B38] Ruano-Rosa D. del Moral-Navarrete L. Lopez-Herrera C. J. (2010). Selection of *Trichoderma* spp. isolates antagonistic to Rosellinia necatrix. Span. J. Agric. Res. 8, 1084–1097. 10.5424/sjar/2010084-1403

[B39] Tello J. Lacasa A. Molina R. (1985). Una nota fitopatológica sobre el complejo parasitario del pie de la judía (*Phaseolus vulgaris*). ITEA 61, 57–69.

[B40] Upchurch R. G. Ramirez M. E. (2010). Defense-related gene expression in soybean leaves and seeds inoculated with *Cercospora kikuchii* and *Diaporthe phaseolorum* var. meridionalis. Physiol. Mol. Plant Pathol. 75, 64–70. 10.1016/j.pmpp.2010.08.007

[B41] Valenciano J. B. Casquero P. A. Boto J. A. Marcelo V. (2006). Evaluation of the occurrence of root rots on bean plants (*Phaseolus vulgaris*) using different sowing methods and with different techniques of pesticide application. N.Z. J. Crop. Horticul. Sci. 34, 291–298. 10.1080/01140671.2006.9514419

[B42] Vellicce G. R. Ricci J. C. D. Hernández L. Castagnaro A. P. (2006). Enhanced resistance to *Botrytis cinerea* mediated by the transgenic expression of the chitinase gene ch5B in strawberry. Transgenic Res. 15, 57–68. 10.1007/s11248-005-2543-6 16475010

[B43] Veloso J. Díaz J. (2012). *Fusarium oxysporum* Fo47 confers protection to pepper plants against *Verticillium dahlia*e and *Phytophthora capsici*, and induces the expression of defence genes. Plant Pathol. 61, 281–288. 10.1111/j.1365-3059.2011.02516.x

[B44] Woo S. L. Lorito M. (2007). Exploiting the interactions between fungal antagonists, pathogens and the plant for biocontrol, in Novel Biotechnologies for Biocontrol Agent Enhancement and Management, eds Vurro M. Gressel J. (Dordrecht: Springer), 107–130.

[B45] Woo S. Scala F. Ruocco M. Lorito M. (2006). The molecular biology of the interactions between *Trichoderma* spp., phytopathogenic fungi, and plants. Phytopathol 96, 181–185. 10.1094/PHYTO-96-018 18943922

[B46] Yedidia I. Srivastva A. K. Kapulnik Y. Chet I. (2001). Effect of *Trichoderma harzianum* on microelement concentrations and increased growth of cucumber plants. Plant Soil 235, 235–242. 10.1023/A:1011990013955

[B47] Zhao Y. Thilmony R. Bender C. L. Schaller A. He S. Y. Howe G. A. (2003). Virulence systems of *Pseudomonas syringae* pv. tomato promote bacterial speck disease in tomato by targeting the jasmonate signaling pathway. Plant J. 36, 485–499. 10.1046/j.1365-313X.2003.01895.x 14617079

